# PET/CT Technology in Adult Zebrafish: A Pilot Study Toward Live Longitudinal Imaging

**DOI:** 10.3389/fmed.2021.725548

**Published:** 2021-10-11

**Authors:** Carl Tucker, Richard Collins, Martin A. Denvir, Wendy A. McDougald

**Affiliations:** ^1^Bioresearch & Veterinary Services (BVS) Aquatics Facility, College of Medicine and Veterinary Medicine, University of Edinburgh, Edinburgh, United Kingdom; ^2^Edinburgh College of Arts, University of Edinburgh, Edinburgh, United Kingdom; ^3^British Heart Foundation (BHF)-Centre for Cardiovascular Science, College of Medicine and Veterinary Medicine, University of Edinburgh, Edinburgh, United Kingdom; ^4^Edinburgh Preclinical Imaging, Edinburgh Imaging, University of Edinburgh, Edinburgh, United Kingdom

**Keywords:** 3D printing, zebrafish, radiotracer [^18^F]FDG, pre-clinical PET CT, longitudinal imaging

## Abstract

Decades of research have confirmed the beneficial and advantageous use of zebrafish (*Danio rerio*) as a model of human disease in biomedical studies. Not only are 71% of human genes shared with the zebrafish many of these genes are linked to human diseases. Currently, numerous transgenic and mutant genetic zebrafish lines are now widely available for use in research. Furthermore, zebrafish are relatively inexpensive to maintain compared to rodents. However, a limiting factor to fully utilising adult zebrafish in research is not the fish but the technological imaging tools available. In order to increase the utilisation of adult zebrafish, which are not naturally transparent, requires new imaging approaches. Therefore, this feasibility study: (1) presents an innovative designed PET/CT adult zebrafish imaging platform, capable of maintaining normal aquatic physiology during scanning; (2) assesses the practical aspects of adult zebrafish imaging; and (3) set basic procedural guidelines for zebrafish imaging during a PET/CT acquisition.

**Methods:** With computer aided design (CAD) software an imaging platform was developed for 3D printing. A 3D printed zebrafish model was created from a CT acquisition of a zebrafish using the CAD software. This model and subsequently euthanised zebrafish were imaged post-injection using different concentrations of the radiotracer [^18^F]FDG with CT contrast.

**Results:** PET/CT imaging was successful, revealing levels as low as 0.01 MBq could be detected in the fish. In the zebrafish imaging post-injection distribution of the radiotracer was observed away from the injection site as well as tissue uptake. Potential preliminary husbandry and welfare guidelines for the fish during and after PET/CT imaging were determined.

**Conclusion:** Using PET/CT for adult zebrafish imaging as a viable non-invasive technological tool is feasible. Adult zebrafish PET/CT imaging has the potential to be a key imaging technique offering the possibilities of enhanced biomedical understanding and new translational data sets.

## Introduction

With zebrafish (*Danio rerio*) as a well-established valuable model to investigate human diseases, continued growth in this field research is expected. In fact, 71% of human genes are associated with one zebrafish orthologue, and of 3,176 human genes with morbidity identification, 82% (2,601) are related to at least one zebrafish orthologue (1). Furthermore, with advancements in targeted genomic mutation tools, such as CRISPR/Cas9, the zebrafish is increasingly utilised for high throughput genotype-phenotyping modelling of human disease has increased (2–5). For example, various zebrafish transgenic lines expressing cell-specific fluorophore markers are available for human neurodegenerative and cardiovascular diseases research, amongst others (6–9). However, most researchers use the embryonic or larval stage where the zebrafish is fully transparent and can be readily imaged using standard light microscope techniques. The challenge arises in the adult where the skin is no longer transparent and where more complex imaging techniques are needed to study organ development or to assess impact of surgical or genetic interventions. *In vivo* imaging of the adult zebrafish is therefore a key topic for further research and development (10, 11).

An important part of this challenge is the need for suitable platforms capable of maintaining fish under physiological conditions during image acquisition over time periods of several hours. Such a platform may also facilitate sequential imaging of the same fish thus allowing longer term assessment of developmental or disease-repair mechanisms over months or even years. Increasing use of the adult zebrafish means that new imaging approaches need to be developed to help support novel translatable research and bridge the imaging gap between existing imaging techniques in the embryo across to the adult.

Positron emission tomography coupled with computed tomography (PET/CT) could provide that bridge by overcoming the hurdles of extending imaging beyond the embryonic stages. Pre-clinical PET/CT is a powerful non-invasive imaging tool already extensively used in rodent pre-clinical research. It supports the investigations and evaluations of underlying biological mechanisms, the physiological processes in a healthy or diseased model. Pre-clinical research areas such as cardiovascular or neurodegenerative diseases, oncology and drug discovery regularly take advantage of PET/CT's fully quantitative functionality (12–20).

This feasibility study addresses an unmet need for advanced imaging in the adult zebrafish using PET/CT. Here, we describe the development and early testing of an aquatic imaging platform to maintain a stable physiological state in adult zebrafish during PET/CT image acquisitions designed to accommodate different commercial scanners with the capability of screening drugs and radiotracers with a modestly high-throughput. The development of non-invasive molecular imaging of adult zebrafish has the potential to substantially improve our understanding of biological pathways and ultimately support the development of novel therapeutic strategies for human disease.

## Materials and Methods

### 3D Printed Imaging Platform

Using the McNeel Rhinoceros version 5.3.2 (Seattle, WA USA) 3D computer aided design (CAD) modelling software an aquatic zebrafish imaging platform, composed of a water chamber with monitoring equipment and two separate holding tanks for dosing and recovery has been designed. Designed concept includes monitoring to ensure correct water flow levels, pressure, salinity, oxygen, pH level, automatic dosing (chemical monitoring: anaesthesia, CT contrast, radiotracers or therapeutics) and temperature for zebrafish sustainability throughout imaging. Multiple water chambers as well as inserts were designed. A prototype of the water chamber was printed on a Stratasys Objet 260 Connex 3D printer (Los Angeles, CA USA), using the acrylic based photopolymer material Vero Clear. Imaging chamber has dimensions of 50.0 mm (length) and a 20.0 mm outer diameter, with internal and external sectioned designs. Printed prototype contains a single outer sectioned for the zebrafish placement. The imaging chamber system is an enclosed flow compartment with environmental monitoring to ensure stable physiology during scanning and a flow pump to circulate water continuously through the system. Dosing and recovery tanks are similarly constructed and environmentally monitored. The dosing chamber is also designed to permit delivery of anaesthetic, CT contrast, radiotracers or other therapeutics within the circulating water. The imaging system includes a designed “bed” holder allowing the platform to sit just beyond the forefront of the scanner bed.

### Zebrafish 3D Printed Model

A CT acquisition of an adult Zebrafish was exported into OsiriX v.7.0 (Bernex, Switzerland). OsiriX's 3D volume and surface rendering tools were used on the FBP reconstructed images, converted from DICOM to standard tessellation language (STL) files and exported into Rhinoceros CAD software. The STL files were then edited to include a simplistic vascular circulatory system (hollow cylinder, radius 0.50 mm), cleaning of unwanted surfaces and artefacts. The prepared CAD files were 3D printed using the Stratasys Objet 260 Connex 3D printer using material Vero Clear. This phantom is dimensionally, geometrically an adult zebrafish with a main simplistic representative circular vascular system. The phantom replaces the use of animals used in determining imaging methods/feasibility, compliant with the National Centre for the Replacement, Refinement and Reduction of Animals in Research (NC3Rs).

### PET/CT Imaging

All images were acquired with the zebrafish imaging platform placed just beyond the frontend of the scanner bed, positioned inside the bore at the isocenter, aligning sagittal, axial and coronal planes. Zebrafish and/or the 3D printed zebrafish models were placed directly inside the water filled imaging platform. CT acquisitions were acquired with the standard protocol (tube voltage 50 kVp, 300 ms, 360 projections) and reconstructed using the filtered back projection (FBP) method with a Cosine filter. Twenty-minute PET acquisitions were acquired in which images were reconstructed using ordered subsets expectation maximisation (OSEM) method with 4 iterations and 6 subsets, with scatter, normalisation and randoms corrections. All PET and CT imaging was done on the Mediso nanoPET/CT (Budapest, Hungary).

#### Preparation of Zebrafish 3D Printed Model

A concentration of 2 ml of water with 0.75 ml of CT contrast agent Ominpaque was prepared for use. Prior to imaging, measured radiotracer ^18^F-deoxyglucose, [^18^F]FDG (0.01–1 MBq) with the CT contrast concentration was injected directly into the designed general simplistic vascular circulatory system using a Hamilton 35-guage needle under a binocular microscope. The phantom was imaged at different times for *n* = 4.

#### Preparation of Zebrafish (Adult Wild Type Wik)

In a series of pilot experiments, adult wild-type zebrafish were first euthanised using Tricaine [Sigma Aldrich, Cat.# A-5040, 4.2%w/v (21)] and the brain destroyed by a modified pithing method using a 15-gauge needle in compliance with the schedule 1 *UK Animals (Scientific Procedures) Act 1986 (ASPA)* prior to placing the animal in the scanner. In this scenario the heart continues to beat for 30–60 min after brain death thus maintaining circulation and the integrity of the fish tissue during the imaging period (22, 23).

An initial activity concentration of 2 mL of water, 65 MBq of radiotracer [^18^F]FDG with 0.75 mL of CT contrast agent Ominpaque was prepared for use. Measured various amounts (0.01–2 MBq) of the activity concentration was injected into the hearts or pectoral cavity of the adult zebrafish using a Hamilton 35-gauge needle under binocular microscope guidance prior to imaging. PET/CT imaging started 30 or 40-min post-injections. Overall *n* = 12 euthanised zebrafish were imaged.

## Results

### 3D Printed Imaging Platform

**Figure 1:**
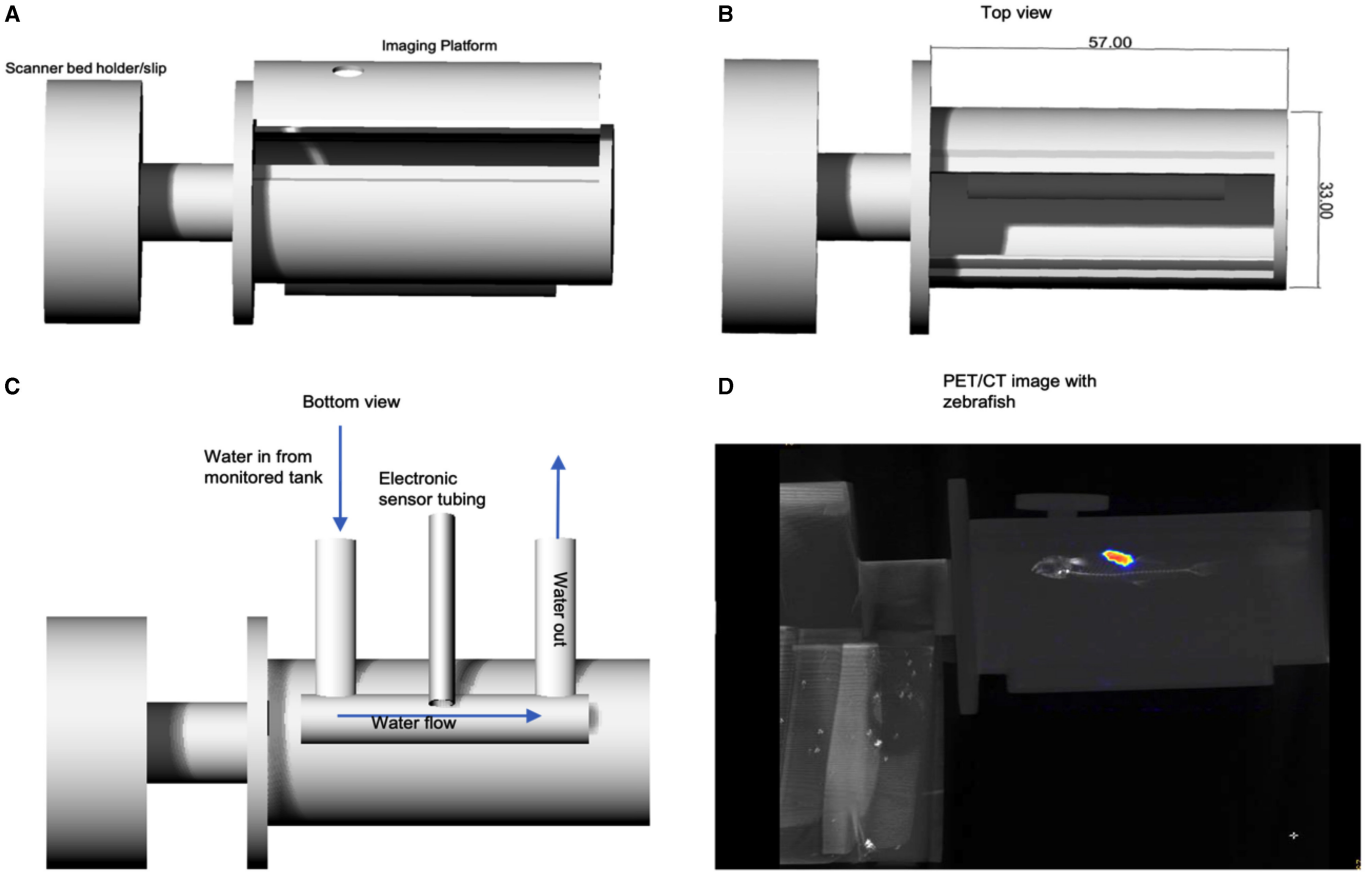
**(A)** Rhinoceros CAD schematic of zebrafish imaging chamber platform prototype set to print for feasibility testing. Bed holder/slip was printed for use on the Mediso nanoPET/CT. **(B)** Top view showing dimensions. **(C)** Displays the design for the environmental monitoring sensor cables and the water in/out flow channel, which run below imaging platform. A reconstructed PET/CT acquisition is shown in **(D)** in which an adult zebrafish can be seen in the imaging platform.

Figure 1 displays the first digital design for the printed prototype chamber used in testing the feasibility of PET/CT imaging of adult zebrafish in a sustainable aquatic imaging platform. This prototype was used in the image acquisition testing of the 3D model zebrafish and for subsequent PET/CT imaging of zebrafish.

### Zebrafish 3D Printed Model

**Figure 2:**
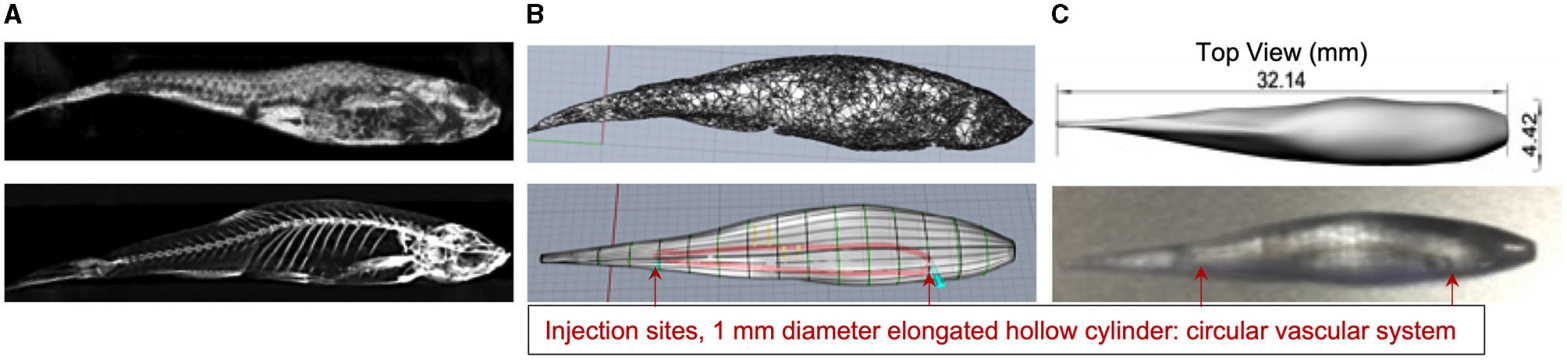
**(A)** CT zebrafish reconstructed images were exported into OsiriX and converted to STL files for exporting into the Rhinoceros CAD software. **(B)** Displays the SLT file (top) and the prepared CAD file for 3D printing (bottom). **(C)** Shows a shaded top view of the CAD file with the fish dimensions (top) and the printed zebrafish model using Vero Clear which included a 1 mm diameter hollow elongated cylinder inside for the injection of CT contrast and radioactivity (bottom).

CT images of two euthanised adult zebrafish were acquired for the designed 3D print a zebrafish model. Figure 2 shows the results of developing the 3D printed zebrafish model; CT acquisition, STL files, CAD preparation, including creating the 1 mm diameter hollow elongated cylinder inside for the injection of CT contrast and radiotracer and (4) the final Vero Clear 3D printed zebrafish model.

### PET/CT Imaging

The fundamental and vital principle of PET/CT imaging lies in the scanner's ability to detect the injected radiotracer. Therefore, various levels of radiotracer where injected to determine the lowest levels suitable for detection in zebrafish imaging. Additionally, a crucial consideration when acquiring pre-clinical PET/CT images is the well-being and welfare of the animals. Establishing the foundations for standard procedures in accordance with the National Centre for the Replacement, Refinement and Reduction (NC3Rs) principles and the Animals (Scientific Procedures) Act 1986 is a critical aspect of studying the feasibility of adult zebrafish PET/CT imaging. At this stage and in conjunction with radiotracer levels, imaging time as well as required radioactive zebrafish recovery times where considered, thus, allowing for the eventual proper fish transportation to the aquatic facility.

#### Zebrafish 3D Printed Model

**Figure 3:**
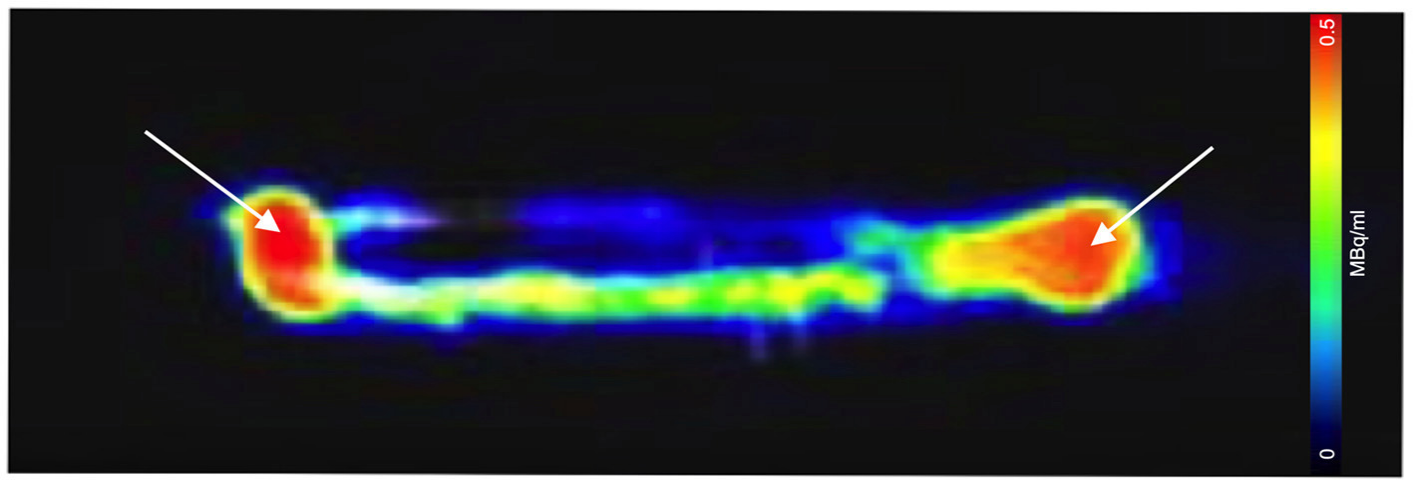
Sagittal PET/CT image acquisition of the 3D printed zebrafish model post-injection of 0.01 MBq of [^18^F]FDG. [^18^F]FDG is seen within the 1 mm simplistic vascular system, though substantial amounts remained at the injection site (white arrows). Adjustments to the injection procedure were made allowing for improve follow in the hollow plastic cavity.

**Figure 4:**
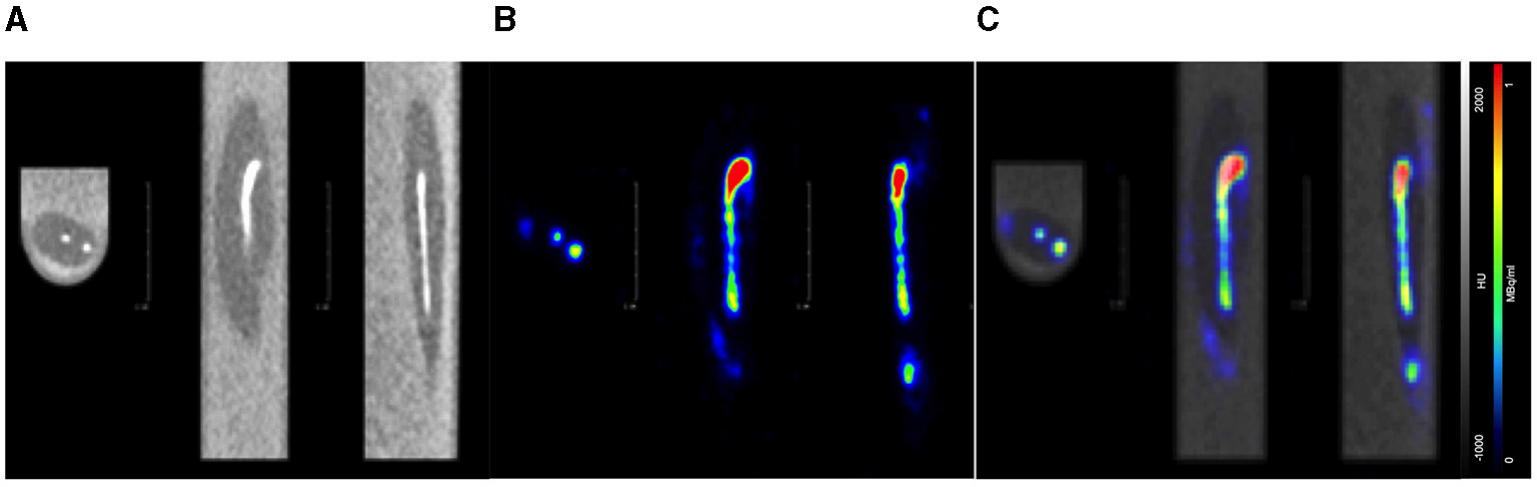
Axial, coronal, and sagittal PET/CT image acquisition of the 3D printed zebrafish model post-injection of 0.05 MBq of [^18^F]FDG. **(A)** CT image displays the CT contrast (white line) withing the model's vascular system. **(B)** PET image reveals [^18^F]FDG (coloured line) within the model's vascular system. **(C)** Fused PET/CT confirms CT contrast and [^18^F]FDG are within the model's vascular system.

A 20-min PET/CT was acquired immediately after the zebrafish 3D model was injected with the CT contrast agent and 0.01 MBq of [^18^F]FDG using the water filled zebrafish imaging platform. Imaging data sets from the 3D printed model indicate that the injected concentration of [^18^F]FDG can be detected within the 0.5 mm radius elongated cylindrical cavity. Figure 3 displays the detection of [^18^F]FDG in the 3D model PET image, though the majority is seen at the injection sites. In Figure 4A the CT contrast is clearly seen in the 3D model's simplistic vascular system. Additionally, in Figure 4B [^18^F]FDG is also detected within the model's vascular system. Figure 4 is a clear indication that a mixture of CT contrast agent and the low level 0.05 MBq of [^18^F]FDG is detectable.

#### Zebrafish (Adult Wild Type Wik)

PET/CT acquisitions of the adult zebrafish were imaged with various amounts of [^18^F]FDG with CT contrast agent, which ranged from 0.01 to 2 MBq. As noted, this was done to assess the viability of the low levels of radiotracer detection as well as determining recovery times for the radioactive zebrafish after imaging for proper husbandry and transportation of the fish (i.e., radioactivity less than background).

**Figure 5:**
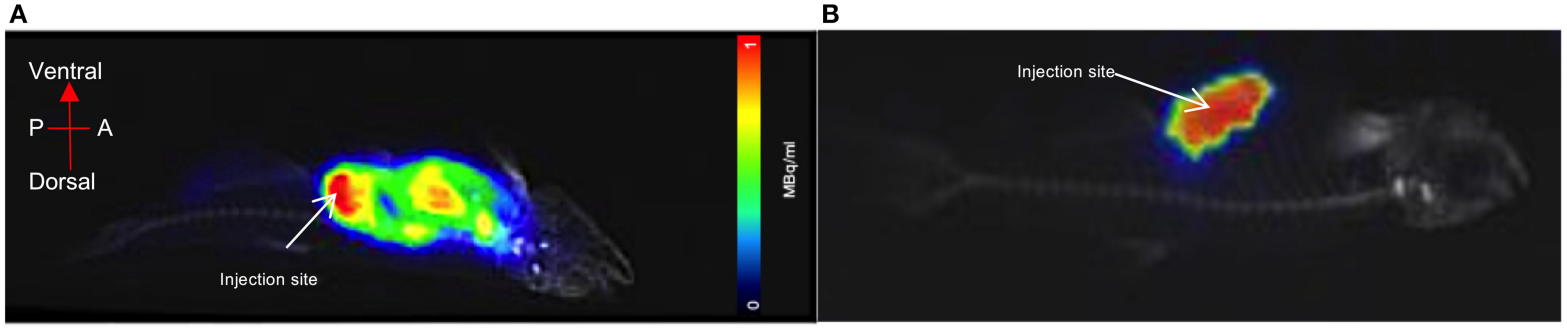
PET acquisition of a euthanized zebrafish inside the imaging platform. **(A)** The mixture of CT contrast and [^18^F]FDG (0.5MBq) was injected into the pectoral cavity immediately following confirmed death and allowed to circulate for 30 min prior to imaging. Distribution and uptake of the activity can be seen away from the injection site. The tracer distribution away from the injection site (red) areas will represent metabolically active organs such as the liver, gut or heart. **(B)** For visual comparison of [^18^F]FDG uptake and distribution, radiotracer has remained at the injection site (pectoral cavity).

A euthanised adult zebrafish, pectoral cavity post-injection with CT contrast agent and 0.43 MBq of [^18^F]FDG, is shown in Figure 5. As noted, all images were acquired as a 20-min PET, a standard CT with the zebrafish placed inside the water filled imaging platform. PET/CT image, shown in Figure 5A, clearly reveals distribution and uptake of the [^18^F]FDG within the zebrafish, with possible uptake in the liver. A full histological analysis has yet to be performed. However, organs demonstrating [^18^F]FDG uptake would be areas representing metabolically active organs including the liver, gut, and heart. For a visual comparison, Figure 5B shows a pectoral cavity post-injection with CT contrast agent and 0.03 MBq of [^18^F]FDG which did not circulate around the zebrafish in the same manner as the other.

## Discussion

We demonstrated that a commercial pre-clinical PET/CT is capable of detecting the common PET radiotracer [^18^F]FDG at low enough dose levels plausible for safe zebrafish imaging and recovery. The lowest dose level detected was 0.01MBq, albeit within the 1 mm diameter 3D printed zebrafish model hollow cylinder, “vascular system,” Figures 3, 4. In the pithed-zebrafish the lowest detectable [^18^F]FDG dose was 0.05 MBq (Figure 5) which confirms that high doses of tracer are not required. A cursory check of [^18^F]FDG uptake, shown in Figure 5, using the maximum standard uptake value (SUV) revealed activity in those selected areas. Extracting a SUV max from the zebrafish images indicates PET quantitative analysis can be accomplished.

This study used the method of direct injection of radiotracer by a Hamilton 35-gauge needle directly into the heart or the pectoral cavity. Therefore, we have not yet tested the method of introducing or treating zebrafish with anaesthesia, CT contrast, radiotracers or therapeutics *via* the circulating water in our physiological chamber. However, potential guidelines for the welfare and recovery of live fish, especially critical for longitudinal studies can be extrapolated from our results. For example, this study would indicate that a dose of 0.5 MBq for a 20-min PET/CT would be sufficient for detection, thus requiring ~4 h of recovery to reach a safe level for transport back to the aquatic facility. It should be noted, (1) radioactivity at background levels or below is considered safe for transportation, based off the International Atomic Energy Agency (IAEA) regulations, (2) the time frame estimated is subject to change based on future work observing the recovery of live zebrafish, adhering to ASPA and the NC3Rs observational guidelines. The current time frame is to establish an initial guideline, which was determined using the decay time of ^18^F and post-measurements of the deceased imaged fish.

In 2013 Koba et al. published proof of concept studies using PET/CT for larger goldfish (40 g) research, thus supporting the feasibility of this research avenue (17). More recently, in 2017 Merrifield et al. developed an aquatic flow cell system capable of monitoring zebrafish during pre-clinical MRI acquisitions (24). In a similar fashion, the PET/CT imaging platform is designed to contain future in/out water flow tubing with electronic sensors for monitoring temperature, pressure, salinity, oxygen, and pH level of the water. Unfortunately, though, routine *in vivo* imaging of adult zebrafish has yet to be established. Now, with greater insight as to the requirements, practicalities of maintaining a suitable dosing, imaging and recovery systems the probability of successfully imaging adult zebrafish increases. Furthermore, our imaging platform design provides the ability for different scanner bed holders/slips to be 3D printed, thus, adopting to different scanners, Figure 1. This approach also allows for the zebrafish to be imaged in the forefront of the scanner bed in order to reduce potential scatter and void attenuation through a scanner bed.

Literature spanning three decades has validated the zebrafish as an excellent model for researching human diseases and drug development. For instance, atherosclerotic disease in zebrafish using [^18^F]FDG, which is the mainstay radioligand in PET imaging and consequently has been the most common radioligand used in imaging studies of atherosclerosis (25, 26). As noted, PET/CT plays a pivotal role in therapeutics and radiotracer development, discovery or repurposing. Results in this capacity have direct translational applications to clinical research and patients. However, from start of research to regulatory approval, a new therapeutic drug typically takes 10–15 years before it reaches the pharmacist. The ability to incorporate live zebrafish PET/CT imaging has the potential to immediately reduce that timescale, by a minimum of 2 years (27). Pre-clinical PET/CT imaging of live zebrafish will assist expeditiously by statistical power/high throughput (large *n* number). This will be achieved by enhancing our understanding of the molecular pathogenesis of disease, increase confidence and success rate when identifying the drug lead compound for future development, and streamlining the validation of the effectiveness of the compound (large *n* numbers for reduced cost compared with alternative pre-clinical species). Therefore, reducing attrition rate for drug candidate selection and characterisation prior to expensive and lengthy human studies, reducing the R&D timescale.

This imaging platform provides the zebrafish research community access to a translatable molecular imaging modality. Thus, shortening research translation time and increasing the relevance of research findings to humans. Additionally, in a cardiovascular context, this adult zebrafish imaging potentially provides newer insights into vascular heart repair mechanisms. Thus, allowing for new approaches to assess novel therapeutic options that could improve recovery of heart function following cardiac injury, such as myocardial infarction.

Researching models of hyperglycaemia in zebrafish, after injecting 20 MBq of [^18^F]FDG in the pectoral cavity Dorsemans et al. (28) successfully acquired a PET/CT noting a wide distribution of [^18^F]FDG. Likewise, in 2017 for proof of concept Snay et al. successfully imaged zebrafish with 0.74–1.29 MBq of [^18^F]FDG, with 10 min PET followed by a 10 min CT acquisition (29). Albeit, in both cases the zebrafish imaging was done with the fish out of water, either in agar or wrapped in a tissue. More recently, for proof of concept Nazario et al. (30) intraperitoneally injected zebrafish with [^18^F]FDG, or ^18^F-NaF, (2–3 MBq) for 5 min PET/CT imaging (30). Here they were able successfully image with the fish placed in a water/tricaine solution filled 15 mL tube. Though not imaged under favourable physiological conditions these recent proof of concepts studies show promise as well as the interest in zebrafish PET/CT imaging.

Nevertheless, continued small fish PET/CT protocol and methodology imaging strategic research is needed in order to fully develop imaging guidelines and best practise procedures. For example, future work will include the use of live zebrafish receiving a concentration of CT contrast, [^18^F]FDG and anaesthetic *via* immersion (inhalation) and tail vein injections. Additionally, recovery procedures will be expanded to include zebrafish physical observations during recovery.

## Conclusion

This pre-clinical PET/CT feasibility study successfully demonstrates the possibility of adult zebrafish imaging using [^18^F]FDG. The development of PET/CT imaging technology to image adult zebrafish has the potential to enhance and expand research in this highly versatile animal model system by extending structural and functional imaging capabilities beyond the embryo.

## Data Availability Statement

The raw data supporting the conclusions of this article will be made available by the authors, without undue reservation.

## Ethics Statement

The animal study was reviewed and approved by the University of Edinburgh Animal Welfare and Ethical Review Board. All experiments were carried out in accordance with the accepted standards of humane animal care under the regulation of the UK Animal (Scientific Procedures) Act 1986 and EU Directive 2010/63/EU. All adult WIK zebrafish were end of life euthanized in accordance with ASPA schedule 1 procedure.

## Author Contributions

WM developed and designed the zebrafish imaging platform, created image acquisition protocols, acquired the 3D model and zebrafish images, carried out the data collection, analysis, and drafted the manuscript. CT assisted in the handling of the zebrafish, interpretation of the data, and the revising of the manuscript. RC assisted in the designing of the zebrafish imaging platform and 3D fish model, 3D printed the platform, and model as well as assisted in the revision of the manuscript. MD assisted in the interpretation of the data and the revising of the manuscript. All authors participated in the conception of the study and design, read, and approved the final manuscript.

## Conflict of Interest

The authors declare that the research was conducted in the absence of any commercial or financial relationships that could be construed as a potential conflict of interest.

## Publisher's Note

All claims expressed in this article are solely those of the authors and do not necessarily represent those of their affiliated organizations, or those of the publisher, the editors and the reviewers. Any product that may be evaluated in this article, or claim that may be made by its manufacturer, is not guaranteed or endorsed by the publisher.
